# Evaluation of Judet view radiographs accuracy in classification of acetabular fractures compared with three-dimensional computerized tomographic scan: a retrospective study

**DOI:** 10.1186/s12891-020-03441-9

**Published:** 2020-06-26

**Authors:** Sepideh Abdi Tazeabadi, Shima Ghafourian Noroozi, Meysam Salehzadeh, Mansour Bahardoust, Hossein Farahini, Mikaiel Hajializade, Ali Yeganeh

**Affiliations:** 1 Resident of Radiology, e Rasoul-e-Akram Hospital, Iran University of Medical Sciences, Tehran, Iran; 2 Department of Radiology, Rasoul-e-Akram Hospital, Iran University of Medical Sciences, Tehran, Iran; 3 Department of Epidemiology, School of Public Health, Iran University of Medical Sciences, Tehran, Iran; 4 Bone and Joint Reconstruction Research Center, Shafa Orthopedic Hospital, Iran University of Medical Sciences, Tehran, Iran; 5 Department of Orthopaedics Surgery, Rasul-e Akram Hospital, Iran University of Medical Sciences, Nyaiesh Ave, Tehran, Iran

**Keywords:** Acetabular fracture, Plain radiograph, Three-dimensional computerized tomographic scan, Judet view

## Abstract

**Background:**

In the current diagnostic procedure, generally, both plain radiographs and 3D-CT scans are used for the diagnosis of acetabular fractures. There is no consensus regarding the value of a three-dimensional computerized tomographic (3D-CT) scan alone in the classification of acetabular fractures. In this study, we compared the accuracy of 3D-CT scan and plain radiography through the evaluation of their agreement with the intraoperative surgeon’s classification.

**Method:**

In a retrospective study, patients who were referred to our center with an acetabular fracture and underwent surgical treatment were included. The classification of acetabular fractures was performed once using Judet view plain radiographs and once using a 3D-CT scan by the corresponding one Experienced musculoskeletal radiologist one independent trauma fellowship-trained orthopaedic who routinely treat acetabular fractures and based on Letournel and Judet classification (17 and 23 years of experience respectively). Cohen’s kappa value was used for the assessment agreement between the two imaging modalities, as well as between the imaging modalities and intraoperative classification.

**Results:**

Medical files of 152 patients with acetabular fracture were retrospectively reviewed. A kappa value of 0.236 was obtained as the agreement level between radiographs and intraoperative findings (*p* < 0.001). A kappa value of 0.943 was obtained as the agreement level between 3D-CT and intraoperative classification (*p* < 0.001). An agreement level of 0.264 was found between the Judet radiographs and 3D-CT scans (*p* < 0.001).

**Conclusions:**

3D-CT scans are reliable enough in the classification of acetabular fractures, and plain radiographs could be omitted to avoid radiation exposure as well as to reduce the cost for patients who sustain acetabular fractures.

## Background

Classification of acetabular fractures is challenging mainly due to the complex three-dimensional (3D) anatomy of the pelvis, as well as the rarity of certain variants of acetabular fracture. Although several systems have been developed to improve the general understanding of these fractures, accurate categorization of acetabular fractures remains a concern for orthopedic surgeons [1, 2]. Yet, the correct classification of acetabular fracture is of the utmost importance to plan the best surgical strategy [3]. Mistyping of acetabular fracture may result in improper treatment and unsatisfactory outcome and even conversion to total hip arthroplasty, which does not necessarily result in a favorable outcome [4, 5].

Judet et al. introduced the radiographic assessment of acetabular fractures using two plain radiographic views of the pelvis, including iliac oblique and obturator oblique views taken with the hip tilted at a 45° angle to the film [6]. Classically, these sets of radiographs were used to classify acetabular fractures [6, 7]. However, the correct classification rate remained very low, so that it showed an accuracy of only 11% when used by inexperienced readers and < 65% when used by general orthopaedists and senior orthopedic residents [8, 9]. Later, 3D-CT imaging modalities emerged to help classification and planning the treatment of acetabular fractures, and preliminary studies revealed considerably improved accuracy of diagnosis following the implication of 3D-CT [3].

In the current diagnostic procedure, generally, both plain radiographs and 3D-CT scans are used for the diagnosis of acetabular fractures [3]. Even so, the impact of this diagnostic approach has not yet been well characterized. There is some evidence showing that 3D-CT alone is accurate enough in the classification of acetabular fracture [9, 10].

In case 3D-CT scans are accurate enough in the diagnosis of acetabular fracture, then plain radiographs could be omitted from the diagnostic process of acetabular fracture, which results in less exposure to ionizing radiation and lower costs for the patients. In addition, positioning patients for Judet view radiographs is generally painful for the patients with pelvic fracture, and even it may cause neurologic injury due to the sharp lip of bone fracture, and omitting these radiographs will also reduce the patient’s pain suffering.

In this study, we aimed to investigate if Judet view radiographs could be eliminated from the diagnostic procedure of acetabular fracture. For this purpose, we compared the agreement level of plain radiographs and 3D-CT scan in the classification of acetabular fractures through the evaluation of the agreement between imaging data with intraoperative findings.

## Methods

This retrospective study was approved by the review board of our institute IR.IUMS.FMD.REC.1398. 390. The patients provided written consent to use their medical data for publication. Between November 2015 and June 2019, consecutive patients who were referred to our center with an acetabular fracture and underwent surgical treatment were evaluated for inclusion. Patients were excluded if they had a history of previous pelvic fracture, low Glasgow Coma Scale (GCS) due to trauma, congruent, and stable hip as well as any other condition that caused conservative treatment. Patients with incomplete imaging were excluded from the study, as well. From a total of 171 patients, 152 patients were identified as eligible for the study.

The classification of acetabular fracture on Judet view plain radiographs and the 3D-CT scan was by the corresponding one Experienced musculoskeletal radiologist one independent trauma fellowship-trained orthopaedic who routinely treat acetabular fractures and based on Letournel and Judet classification (17 and 23 years of experience respectively) [6]. Accordingly, the fractures were classified into 10 categories (elementary and associated) including anterior wall fracture, anterior column fracture, posterior wall fracture, posterior column fracture, transverse fracture, T-shaped fracture, anterior column or wall with posterior Hemi-transverse fracture, both column fracture, posterior column and posterior wall fracture, and transverse & posterior wall fracture [11].

The CT scans were obtained by using a (TOSHIBA_MEC Activion 16 slice, Japan) with MA = 100, KV = 120, FOV = 390.6, WW = 1500, WL = 500, 5-mm thickness and pitch of 0.6–1.5.

The Judet radiographs comprised of two views, the iliac oblique view for the assessment of the posterior column and anterior wall of the acetabulum and the obturator oblique view for the assessment of anterior column and posterior wall of the acetabulum. For both Judet view radiography, the patients were positioned supine. In the iliac oblique view, the unaffected side was rotated roughly 45° anterior, whereas, in the obturator oblique view, the affected side was rotated roughly 45° anterior.

We first evaluated the agreement level of each imaging modality (radiography and 3D-CT scan) with the intraoperative assessment of fracture type. Then we evaluated the agreement level of two imaging modalities with each other. First, radiographs and then 3D-CT scans were evaluated consecutively. Radiographs and then 3D-CT scans were. Radiographs and then 3D-CT scans newly read. All the intraoperative assessments were done by one senior hip surgeon who independent and blinded from a surgeon and radiologist who classifies fractures.

### Statistical analysis

SPSS for Windows, version 16 (Chicago, Illinois, USA) was used for statistical evaluation of the data. Cohen’s kappa coefficient test was used for the assessment agreement level between the different imaging modalities, as well as between the imaging modalities and intraoperative findings. In this respect, kappa values were classified into five categories including “poor agreement” (> 0.00), “slight agreement” (0 to 0.20), “fair agreement” (0.21 to 0.40), “moderate agreement” (0.41 to 0.60), “substantial agreement” (0.61 to 0.80) and “almost perfect agreement” (> 0.8) [12]. A *p*-value of fewer than 0.05 was considered significant.

## Results

In this study, medical files of 152 patients with acetabular fracture were retrospectively reviewed. The study population included 123 (80.9%) males and 29 (19.1%) females with the mean age of 38.7 ± 13.4 years. The mechanism of injury was car accidents in the majority of patients (113 patients, 74.3%). According to the intraoperative investigations, both column fractures were the most frequent pattern of acetabular fracture (43 patients, 28.3%). The characteristic features of patients and fractures are demonstrated in Table 1.

**Table 1:** The characteristic features of patients and fractures

Variable	Mean ± SD or Number (%)
**Sex**	
• **Male**	123 (80.9%)
• **Female**	29 (19.1%)
**Age (year)**	38.78 ± 13.42
**Mechanism of injury**	
• **Car accident**	113 (74.3%)
• **Falling from height**	31 (20.4%)
• **Contact sport**	8 (5.3%)
**Fracture pattern**	
• **Anterior column**	19 (12.5%)
• **Anterior column/wall with posterior hemitransverse**	3 (2%)
• **Both column**	43 (28.3%)
• **Posterior column**	10 (6.6%)
• **Posterior column & posterior wall**	5 (3.3%)
• **Posterior wall**	38 (25%)
• **T-shaped**	5 (3.3)
• **Transverse**	10 (6.5%)
• **Transverse and posterior wall**	19 (12.5%)

Fracture pattern on Judet radiographs agreed with intraoperative fracture type in 52 (34.2%) cases. Based on these results, a kappa value of 0.236 was obtained as the agreement level between radiographs and intraoperative findings (*p* < 0.001). In 100 cases, the fracture pattern on the Judet radiographs was not consistent with the intraoperative fracture pattern (Table 2).

**Table 2:** Acetabular fracture pattern on Judet radiographs and intraoperative findings

Fracture pattern	Intraoperative finding	Judet radiographs	Kappa value
• **Anterior column**	19 (12.5%)	28 (18.8%)	
• **Anterior column/wall with posterior hemitransverse**	3 (2%)	16 (10.3)	
• **Both column**	43 (28.3%)	9 (6%)	
• **Posterior column**	10 (6.6%)	13 (8.5%)	
• **Posterior column & posterior wall**	5 (3.3%)	0 (0%)	0.236
• **Posterior wall**	38 (25%)	39 (25.8%)	
• **T-shaped**	5 (3.3)	12 (7.9%)	
• **Transverse**	10 (6.6%)	27 (17.8%)	
• **Transverse and posterior wall**	19 (12.4%)	8 (4.9%)	

Fracture patterns on 3D-CT scans agreed with intraoperative fracture type in 144 (94.7%) cases. Accordingly, a kappa value of 0.943 was obtained as the agreement level between 3D-CT and intraoperative classification (*p* < 0.001). In eight cases, the fracture pattern on the 3D-CT scan was not consistent with the intraoperative fracture pattern (Table 3).

**Table 3:** Acetabular fracture pattern on 3D CT scans and intraoperative findings

Fracture pattern	Intraoperative finding	3D CT findings	Kappa value
• **Anterior column**	19 (12.5%)	19 (12.5%)	
• **Anterior column/wall with posterior hemitransverse**	3 (2%)	4 (2.5)	
• **Both column**	43 (28.3%)	43 (28.3%)	
• **Posterior column**	10 (6.6%)	9 (5.6%)	
• **Posterior column & posterior wall**	5 (3.3%)	2 (1.2%)	0.943
• **Posterior wall**	38 (25%)	38 (25.8%)	
• **T-shaped**	5 (3.3)	5 (3.3%)	
• **Transverse**	10 (6.5%)	13 (8.3%)	
• **Transverse and posterior wall**	19 (12.5%)	19 (12.5%)	

**Fig. 1:**
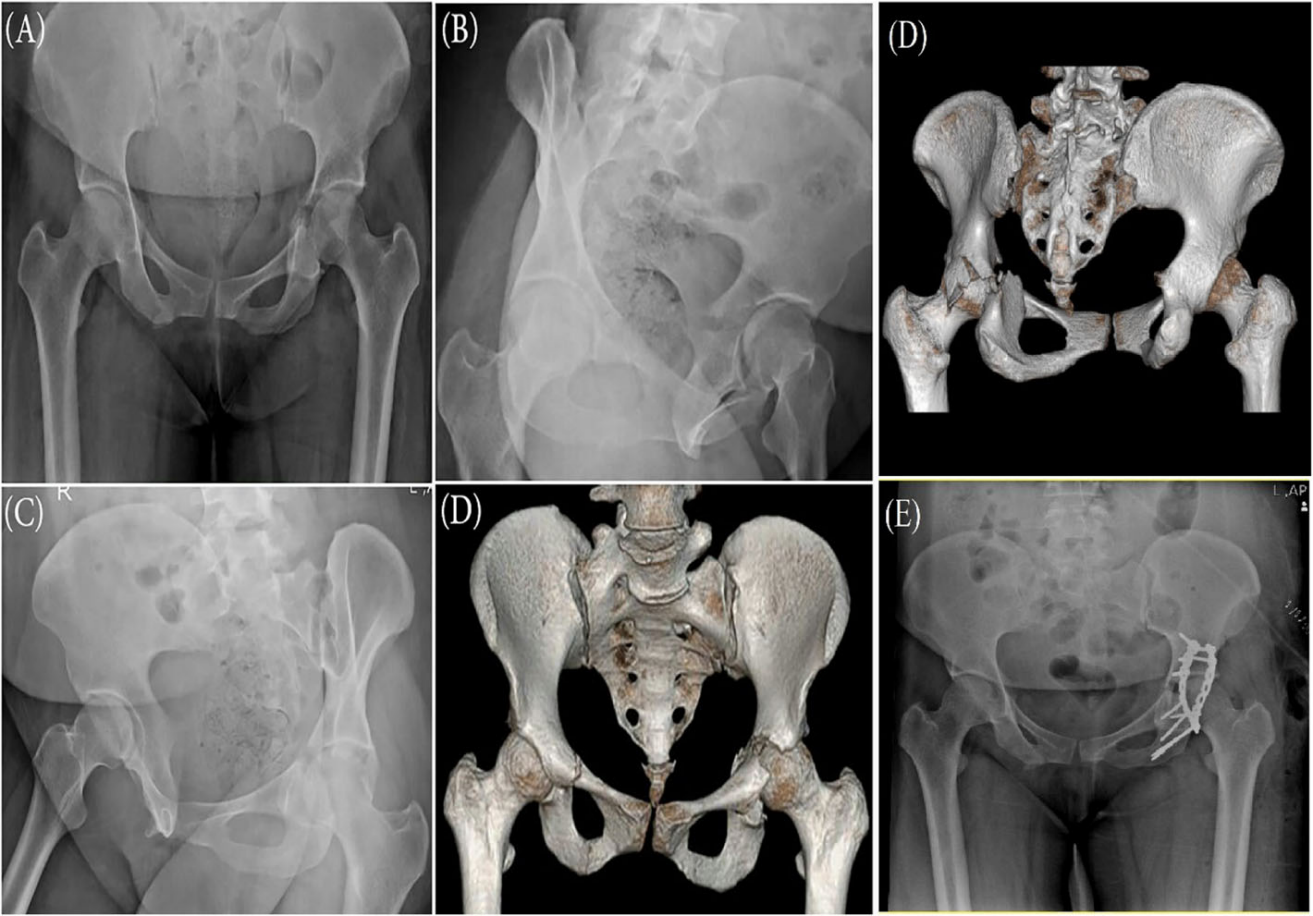
Plain radiographs, **a** Anteroposterior, **b** iliac oblique view, **c**: obturator oblique view. **d** 3D CT-Scan of a patient with the acetabular fracture. The fracture type was reported as transverse in Ap and Judet view radiographs and after 3d ct scan reported as Transverse & posterior wall fracture that changed the plan and approach of surgery. **e** postoperative x-ray showing post wall and column fixation with Kocher approach and caused anterior indirect reduction that don’t need surgery

Fracture pattern on Judet radiographs agreed with fracture pattern on 3D-CT scans in 58 (38.1%) cases. Accordingly, an agreement level of 0.264 was found between the Judet radiographs and 3D-CT scans (*p* < 0.001) (Table 4) (Fig. 1).

**Table 4:** Acetabular fracture pattern on 3D CT scans and Judet radiographs

Fracture pattern	3D CT findings	Judet radiographs	Kappa value
• **Anterior column**	19 (12.5%)	28 (18.8%)	
• **Anterior column/wall with posterior hemitransverse**	4 (2.5)	16 (10.3)	
• **Both column**	43 (28.3%)	9 (6%)	
• **Posterior column**	9 (5.6%)	13 (8.5%)	
• **Posterior column & posterior wall**	2 (1.2%)	0 (0%)	0.264
• **Posterior wall**	38 (25.8%)	39 (25.8%)	
• **T-shaped**	5 (3.3%)	12 (7.9%)	
• **Transverse**	13 (8.3%)	27 (17.8%)	
• **Transverse and posterior wall**	19 (12.5%)	8 (4.9%)

## Discussion

In this study, we aimed to investigate the value of radiography and 3D-CT scan in the accurate diagnosis of acetabular fracture type through comparison of fracture type on imaging modalities with the intraoperative fracture type. Based on our results, Judet radiographs demonstrated a fair agreement with intraoperative fracture pattern (kappa value = 0.236), whereas 3D-CT scans revealed a perfect agreement with intraoperative findings (Kappa value = 0.943). The agreement level between Judet radiographs and 3D-CT scans was fair, as well (Kappa value = 0.264).

Beaulé et al. aimed to study the interobserver and intraobserver reliability of Letournel’s acetabular fracture classification as well as to investigate the effect of CT on its reliability. The images were observed by three groups of orthopedic surgeons and twice, once based on radiographs only and once in combination with CT scan. In the first session, the interobserver reliability with and without CT was 0.74 and 0.7, for group 1, 0.69, and 0.71 for group 2, and 0.51 and 0.51 for group 3, respectively. The results were similar in the second session. The intraobserver reliability (comparison of first and second session) with and without CT was 0.83 and 0.8 for group 1, 0.80, and 0.80 for group 2, and 0.69 and 0.64 for group 3, respectively. They concluded that CT scans do not appear to be essential for the classification of acetabular fractures [13]. Although these results were not consistent with our results, it should be noticed that axial view CT scans and not 3D-CT scans were used in this study.

Visutipol et al. studied the intraobserver reproducibility and interobserver reliability of acetabular fracture typing 20 patients. The images were evaluated by five observers. Based on their study, intraobserver reproducibility of the plain radiographs and 3D-CT scan were 0.42 and 0.44, respectively. Interobserver reliability was 0.24 in both groups. According to these results, they concluded that the 3D-CT scan does not improve either the interobserver reliability or the intraobserver reproducibility in classifying the acetabular fracture [14]. By contrast to the results of this study, the results of the present study reveal a significant improvement in the reliability of acetabular fracture typing using 3D-CT scan. This inconsistency could be attributed to the design of the study. While Visutipol et al. compared the reliability of two imaging modalities with each other, we assessed the reliability of each imaging modality in comparison with the intraoperative surgeon’s classification.

Ohashi et al. retrospectively evaluated the interobserver agreement for acetabular fracture classification using radiography alone and 3D-CT alone in 101 imaging studies from 99 patients. The images were reviewed and classified by two musculoskeletal radiologists independently. The interobserver agreement level was 0.42 with radiography and 0.70 with 3D-CT. After standard Judet views were added, 3D-CT based classification changed in two cases. In surgically treated patients, the agreement level with the surgeons’ classification was higher with 3D-CT than with radiography. They concluded that standard Judet pelvic radiographs add little information for changing the 3D-CT classification [15]. Similar to the study of Ohashi et al., the results of the present series revealed a higher agreement level between the surgeons’ classification and 3D-CT than with radiography.

O’Toole et al. assessed whether the 3D-CT scan improves the acetabular fracture classification in comparison with plain radiographs. Four different image sets from 75 acetabular fractures were.

Evaluated in this study including Judet view plain radiographs plus axial view CT scans (image set A), Judet view plain radiographs alone (image set B), 3D-CT reconstructions (image set C), and CT simulated anteroposterior and Judet views of the pelvis (image set D). In comparison with the gold standard diagnostic method (intraoperative report), plain radiographs alone (image set B) had the worst agreement level. They concluded that 3D-CT scans improve accuracy in classifying the acetabular fracture patterns. These results were in accordance with the results of the present studies [10].

In addition to plain radiographs, axial CT scans using reconstructions and multiplane views are usually used by radiologists and orthopedic Surgeon for fractures evaluation and also classification. The 3D-CT are generated by reformatting the axial images, so why not use the axial CT instead of 3D-CT, as there is no radiation or scan time added? The study of Kickuth et al. revealed that 3D CT was significantly better than axial CT in the classification of the acetabular fractures by surgeons [16]. Therefore, we believe that 3D-CT could also replace axial-CT evaluation. Because it gives us a real view of fracture instead of Spatial visualization of fracture in the mind.

Although the value of 3D-CT in the classification of acetabular fracture has also been discussed in several other investigations [2, 3, 17–21], the effect size of this imaging modality in optimizing the acetabular fracture typing is still unclear. According to the results of the present study, 3D-CT alone is reliable enough acetabular fracture typing, and plain radiograph could be omitted from this classification.

The present study was not without limitations. Due to the retrospective identity of research, we did not evaluate inter- and intraobserver reliability of our evaluations, which could be regarded as the main limitation of the study. While our results revealed the substantial agreement between 3D-CT and the surgeon’s classification, it is not clear whether these results could be repeated with different observers or by the same observer over time. Therefore, this limitation should be avoided in future investigations.

## Conclusion

While 3D-CT scans revealed a substantial agreement with intraoperative surgeon’s classification of acetabular fracture, Judet view radiographs demonstrated a small agreement. These results suggest that 3D-CT scans are reliable and clinically efficacious in the classification of acetabular fractures, and plain radiographs could be omitted to avoid radiation exposure as well as to reduce the cost and pain for patients who sustain acetabular fractures.

## Data Availability

The datasets used and analyzed during the current study are available from the corresponding author on reasonable request.

## Abbreviation

_ 3D-CT: Three-Dimensional Computerized Tomographic
